# The effect of breast density on the missed lesion rate in screening digital mammography determined using an adjustable-density breast phantom tailored to Japanese women

**DOI:** 10.1371/journal.pone.0245060

**Published:** 2021-01-07

**Authors:** Mika Yamamuro, Yoshiyuki Asai, Naomi Hashimoto, Nao Yasuda, Yoshiaki Ozaki, Kazunari Ishii, Yongbum Lee

**Affiliations:** 1 Radiology Center, Kindai University Hospital, Osaka-sayama, Osaka, Japan; 2 Graduate School of Health Sciences, Niigata University, Niigata, Japan; 3 Research Institute of Scientific Investigation, Kyoto Prefectural Police Headquarters, Kyoto, Japan; 4 Kindai University Faculty of Medicine, Osaka-sayama, Osaka, Japan; Medical University of Vienna, AUSTRIA

## Abstract

**Objective:**

Despite the high risk of missing lesions in mammography, the missed lesion rate is yet to be clinically established. Further, no breast phantoms with adjustable breast density currently exist. We developed a novel, adjustable-density breast phantom with a composition identical to that of actual breasts, and determined the quantitative relationship between breast density and the missed lesion rate in mammography.

**Methods:**

An original breast phantom consisting of adipose- and fibroglandular-equivalent materials was developed, and a receiver operating characteristic (ROC) study was performed. Breast density, which is the fraction by weight of fibroglandular to total tissue, was adjusted to 25%, 50%, and 75% by arbitrarily mixing the two materials. Microcalcification, mass lesions, and spiculated lesions, each with unique characteristics, were inserted into the phantom. For the above-mentioned fibroglandular densities, 50 positive and 50 negative images for each lesion type were used as case samples for the ROC study. Five certified radiological technologists participated in lesion detection.

**Results:**

The mass-lesion detection rate, according to the area under the curve, decreased by 18.0% (*p* = 0.0001, 95% Confidence intervals [CI] = 0.1258 to 0.1822) and 37.8% (*p* = 0.0003, 95% CI = 0.2453 to 0.4031) for breast densities of 50% and 75%, respectively, compared to that for a 25% breast density. A similar tendency was observed with microcalcification; however, spiculated lesions did not follow this tendency.

**Conclusions:**

We quantified the missed lesion rate in different densities of breast tissue using a novel breast phantom, which is imperative for advancing individualized screening mammography.

## Introduction

Breast cancer is one of the most common cancer types affecting women around the world [1–4], and early detection is required in order to decrease the high mortality rate. Full-field digital mammography (FFDM) is a two-dimensional imaging modality that is widely used for breast cancer screening. FFDM is the sole modality for which there is evidence of decreasing mortality rate of approximately 20% [5], and it is essential as a screening tool [6–8]. Breast density is an important factor in screening mammography that has a strong impact on lesion detection and breast cancer incidence [9]. In some cases, a dense breast leads to substantial missing of lesions, because of the masking effect of normal fibroglandular tissue [10–15]. In consideration of this limitation, it has become a common practice to notify examinees’ of their breast densities in the USA, spreading to all states by 2019, and further screening with a suitable second modality such as breast echo is recommended based on individual’s breast density [16–20]. Japanese women ranging from 40 to 69 years are invited to undergo a mammographic screening every alternate year. Although Japan is also currently considering notifying examinees when they have a high breast density [21], no quantification algorithm complying with the Breast Imaging Reporting and Data System (BI-RADS) has been determined.

Recently, volumetric breast density measurement (VBDM) using FFDM has been developed, providing a quantitative estimate of breast density. A new method for accurate determination of breast density, using the pixel value generated by FFDM, was reported in a recent paper [22]. However, the relationship between breast density and the risk of missing lesion in screening mammography has still not been clearly established. Chiu et al., in a review describe that “very few studies have elucidated the effect of mammographic density measured at baseline (prediagnostic mammograms) on incidence, stage, mortality, and mammography screening sensitivity related to masking effects using very long follow-up data” [23]. To our knowledge, there have been no studies showing consistent results with respect to the relationship between breast density and the risk of missing a lesion in screening mammography. Ekpo et al. show that the percentage decrease of mammographic sensitivity of dense breasts to fatty breasts vary from 22.1% to 70.0% among previous studies [24]. This is by no means a consistent result. In order to improve individualized screening mammography, the risk of missing a lesion via FFDM for different levels of breast density should be clearly quantified.

To date, the occurrence of interval cancers has been used to estimate the missed lesion rate in clinical screening mammography [25–27], under the assumption that it reflects a lesion that was missed during the previous screening mammography. This assumption, however, does not strictly hold up, as the true onset of interval cancer is unknown. In addition, this method can only be used for women who undergo regular screening. Hollingsworth pointed out that it is not appropriate to use interval cancer as an indicator of mammographic sensitivity [28].

On the other hand, many factors in addition to the breast density, such as distribution of fibroglandular tissue, imaging techniques such as position settings and compressed pressure, compressed breast thickness, patient’s age, reader’s concentration, prevalence rate in population, image processing conditions, etc. are intricately intertwined into screening mammographic sensitivity [24]. As a result, the overall missed lesion rate varies over a wide range as described above. In order to improve the overall missing lesion in clinical screening mammography, an approach by separating individual factors is required. Accordingly, we focused only on the impact of breast density excluding other factors by conducting a phantom study, since a constant condition associated with the other factors could not be practiced by an actual clinical study.

However, most of the commonly used breast phantoms, e.g., polymethyl methacrylate [29,30] phantoms and those produced by Computerized Imaging Reference System, Inc., [31] do not have identical X-ray energy absorption efficiencies over the entire mammographic X-ray energy range. This is because the composition differs with that of the actual breast tissue; accordingly, mammographic image characteristics, such as contrast, differ from that of real breast tissue of the same density. To enable individualized screening mammography, the absorption efficiency of a breast phantom should be consistent over the mammographic X-ray energy range. This will allow the radiologist to estimate accurately the missed lesion rate per examinee, based on individual breast density. Therefore, we have developed a novel adjustable-density breast phantom (hereafter abbreviated as original phantom) having identical X-ray attenuation characteristics over the mammographic X-ray energy range with actual breasts.

Japanese women often have thin breasts and a high breast density compared with those of women of Western countries. For example, the proportion of heterogeneously or extremely dense breast in Dutch woman is 32.5% to 45.7% between regions [32], whereas that in Japanese women is approximately 60% [33]. In addition, Young et al. [34] reported that the average compressed breast thickness (CBT) in 16505 British women is 56.8 mm, whereas Nishide et al. reported a mean compressed breast thickness (CBT) of 31.7 mm for 7566 Japanese women who underwent mammography at Fukui Prefectural Hospital, and 44.8% of those women had a CBT of less than 30 mm [35]. It is well known that breast density tends to increase with decreasing CBT [22,36]. On investigating the distribution of the breast density in Japanese women, it was observed that the thinner the breast thickness, the higher the breast density, e.g. the breast density at 20 mm of CBT in Japanese women is 1.36 times higher than that at 30 mm with high missing lesion risk [22]. Thus, an investigation on missed lesions in Japanese women is required using a phantom modelling drastic thin breast. In this study, we aimed to determine the reliable quantitative relationship between breast density and the missed lesion rate using receiver operating characteristic (ROC) examination [37] using an actual 20-mm-thick breast.

## Materials and methods

### Variable-density breast phantom

Ethics committee approval was waived by Faculty of Medicine Kindai University Ethics Committee because this was a phantom study.

To make an original phantom, adipose- (C: 72.0%, O: 16.4%, H: 9.2%, N: 2.4%) and fibroglandular-equivalent (C: 69.5%, O: 17.3%, H: 8.9%, N: 2.3%, Ca: 1.4%, P: 0.6%) compositions (base resins for making the phantom) were developed in cooperation with Kyoto-kagaku Co., Japan. These compositions were similar to those of the International Commission on Radiation Units and Measurements (ICRU) report 44 [38]. The adipose-equivalent tissue was processed into square slabs of 120 mm × 120 mm, with a thickness of 5 to 30 mm at 5-mm intervals (for the purposes of this study, we only used slabs up to a maximum thickness of 15 mm, because it is the thickness to create the breast densities of 25%, 50%, and 75% in a total breast thickness of 20 mm as combined with fibroglandular-equivalent tissue). The fibroglandular-equivalent tissue was processed to contain branch structures with a diameter of 0.3 mm in minimum to 2.0 mm in maximum each. The manufacturing technique of these tissues was as follows: ① The base resins of adipose-equivalent composition and fibroglandular-equivalent composition were independently weighed in units of 0.1 gram using an electric balance with an activator agent. ② After stirring sufficiently, the resin was degassed using a vacuum defoamer. ③ The resin was poured into a special mold, and left to stand for 24 hours. ④ The hardened resin was removed from the mold, and shaped. All the above steps were carried out in a constant room condition adjusted to a humidity of 40% and a temperature of 23°C.

**Fig 1:**
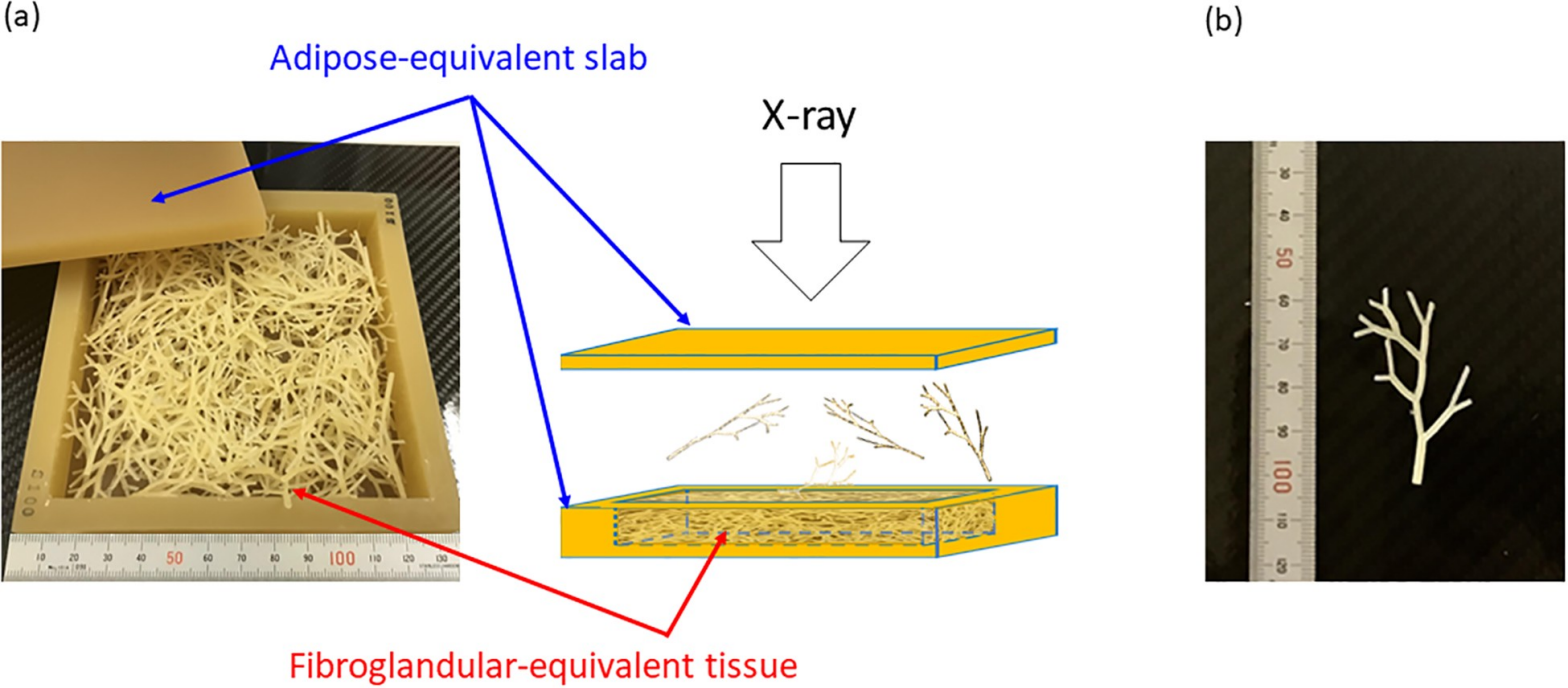
Illustrations of original breast phantom. (a) Assembled breast phantom (b) a piece of fibroglandular-equivalent tissue.

The assembling of the original breast phantom is as follows: ① Using the adipose-equivalent slabs, a box with a cubic-capacity of 120 mm (width) × 120 mm (depth) × 10 mm (height) was made. ② Fibroglandular-equivalent tissues were enclosed into the adipose-equivalent box. These steps are illustrated in Fig 1. In these steps, the thickness of the adipose-equivalent slabs and the amount of fibroglandular-equivalent tissues can be changed arbitrarily. The amount and ratio of fibroglandular- and adipose-equivalent tissues used to generate breast densities of 25%, 50%, and 75% are indicated in Table 1, where breast density refers to the weight ratio of fibroglandular tissue to total tissue.

**Table 1:** Structure of the original phantom used in this study.

Breast density (%)	Adipose-equivalent tissue			Fibroglandular-equivalent tissue			Total	
	Area (mm)	Thickness (mm)	Weight (g)	Area (mm)	Thickness (mm)	Weight (g)	Thickness (mm)	Weight (g)
**25**	120 × 120	15.0	216.0	120 × 120	4.8	72.0	19.8	288.0
**50**	120 × 120	10.0	144.0	120 × 120	9.5	144.0	19.5	288.0
**75**	120 × 120	5.0	72.0	120 × 120	14.3	216.0	19.3	288.0

**Fig 2:**
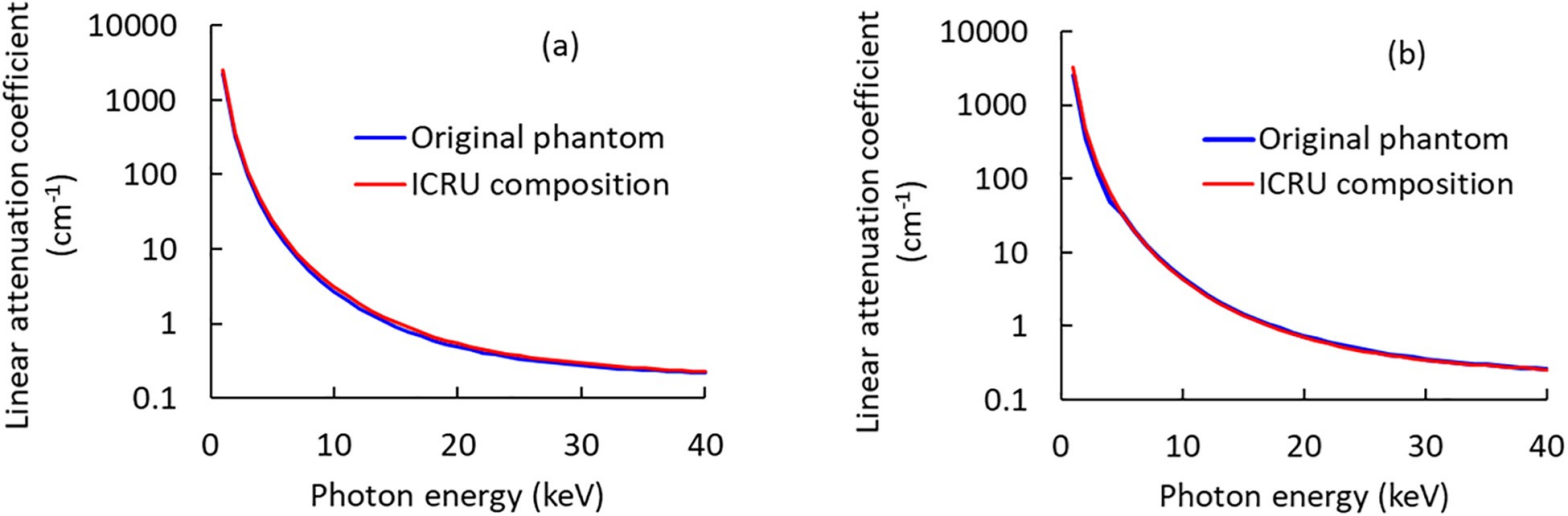
Linear attenuation coefficients of (a) adipose and (b) fibroglandular tissues.

**Fig 3:**
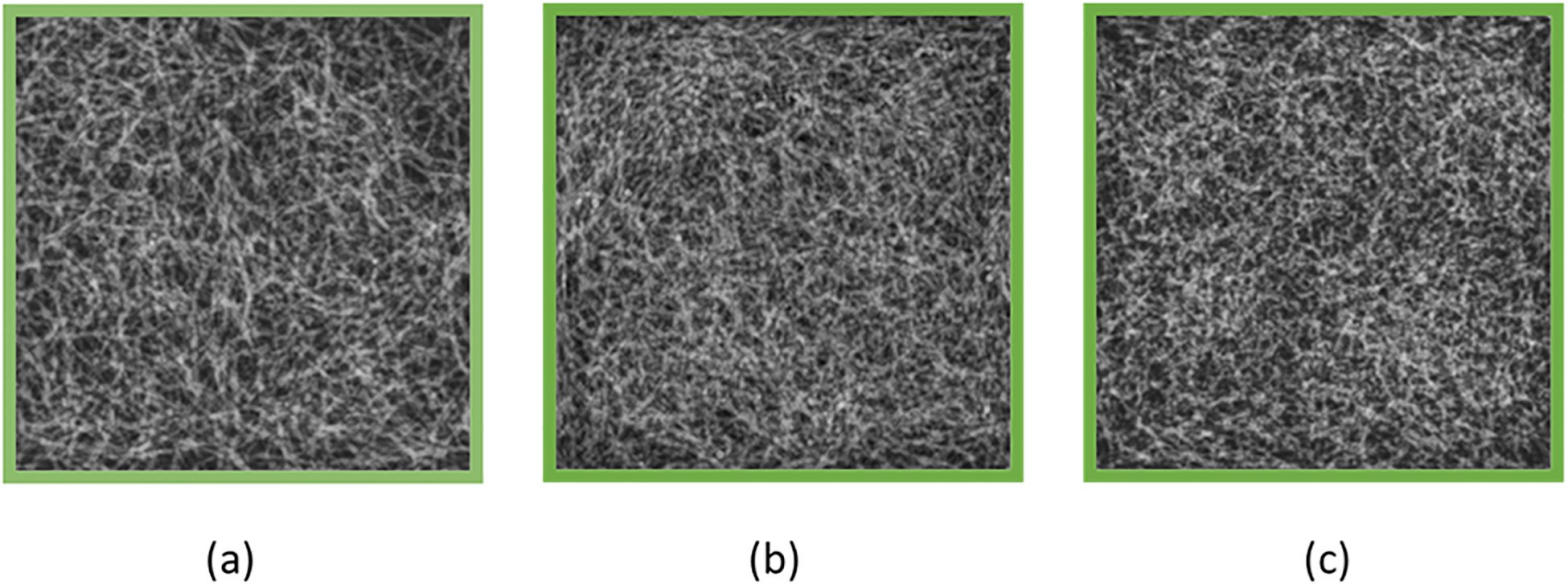
X-ray images of the original phantom. (a), (b), and (c) correspond to breast densities of 25%, 50%, and 75%, respectively.

To confirm the X-ray attenuation characteristics of both the adipose- and fibroglandular-equivalent tissues used for the original phantom, the linear attenuation coefficients of these materials were calculated for the original, and the ICRU compositions using XCOM [39]. The linear attenuation coefficients of the original phantom, and ICRU compositions were in good agreement for both the adipose and fibroglandular tissues over the entire mammographic X-ray energy range (Fig 2). We, therefore, considered the original phantom as identical to actual breast tissue for the purposes of VBDM. Fig 3 shows X-ray images of the original phantom adjusted to breast densities of (a) 25%, (b) 50%, and (c) 75%.

### Receiver operating characteristic study

**Fig 4:**
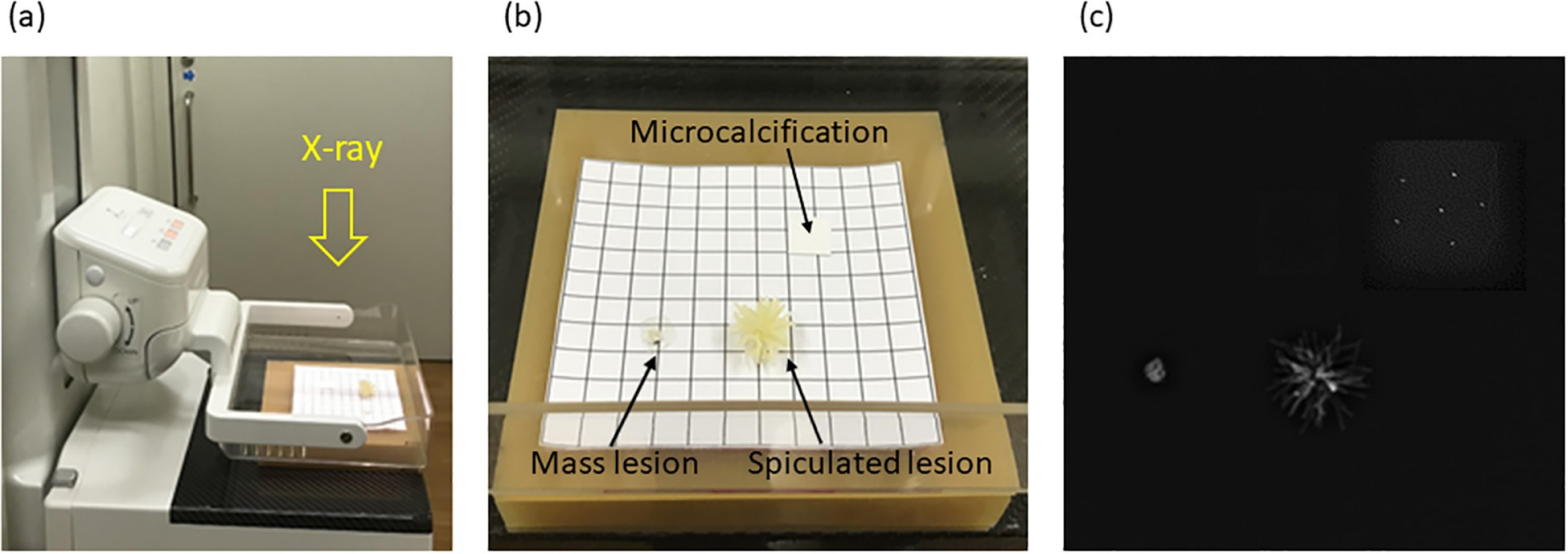
Preparation of receiver operating characteristic (ROC) practice. (a) Mammographic setting (b) An example of lesion placement (c) X-ray image of lesions corresponding to (b). Only microcalcifications are shown with magnification since actual size is only 100 μm in diameter.

Three types of simulated lesions–six each of microcalcifications (calcium carbonate) of 100 μm in diameter, mass lesions of approximately 5 mm in diameter (polyvinyl chloride resin), and spiculated lesions (identical material to that of fibroglandular-equivalent tissue) of 10 mm in diameter–were put on the original phantom in order to study the missed lesion rate. The placement of each lesion was determined by random-number generation after dividing the slab surface into 144 (10 mm^2^) equal parts. Fig 4 shows the geometry of the simulated lesions and the X-ray image without the fibroglandular-equivalent tissue, because of clarification. An ROC study was performed using the original phantom, to determine the effect of breast density on the missed lesion rate. For each lesion type, and for each of the three different breast densities, 50 positive and 50 negative images were used as case samples. All images were radiographed with a Pe-ru-ru digital mammographic system with a flat panel detector (Canon, Tokyo, Japan), equipped with a molybdenum/rhodium target/filter. The pixel size was 75 × 75 μm and the output grey level was 13 bits. Constant exposure conditions were the same as for typical clinical use: a tube voltage of 29 kV, and a tube current of 18.0 mA.

Five certified radiological technologists in screening mammography participated in this study. They were classified into four levels according to certified experience: junior, as less than 1 year of certified experience; semi-senior, as 1 to 5 years of certified experience; senior, as 6 to 15 years of certified experience; and expert, as over 15 years of certified experience. A 21-inch monochromatic liquid crystal display monitor with 5 megapixels and 600 cd/m^2^ luminance was used for observation. Observing conditions were as follows: room illuminance was 20 lx, and no restrictions were placed on observation time and distance between the observer and the image; however, a time limit of two hours was set for sequential observation for each breast density level, to avoid eyestrain. Confidence was assigned for the presence or absence of a lesion, according to a five-point scale: (1), very unlikely to be a lesion; (2), probably not a lesion; (3), possibly a lesion; (4), probably a lesion; (5), definitely a lesion. Statistical analysis was performed using Web-based Calculator for ROC Curves (Eng and Morgan, Department of Radiology and Radiological Science, Johns Hopkins University School of Medicine, Baltimore, Maryland, USA) [40].

To determine whether there was a statistically significant difference between the detectability of lesions for different breast densities, Student’s t test (paired two-tailed) [41,42] was performed on the area under the curve (AUC) using Microsoft Excel for Windows ver. 2016 (Microsoft Corporation, Redmond, Washington, USA). The AUC equals the probability, that the detectability test results from a randomly selected pair of lesion and non-lesion individuals are correctly assigned. In this study, a P value of less than 0.05 was considered statistically significant.

### Noise-power spectrum measurement

To understand the effect of spatial frequencies of the simulated lesions on lesion detectability, the noise-power spectrum (NPS) of the original phantom was measured for 25%, 50%, and 75% breast densities using a two-dimensional fast Fourier transform (2D-FFT) method [43], where ‘noise’ means the distribution of fibroglandular-equivalent tissue in the original phantom. Sub-images of 1024 × 1024 pixels were extracted from the central region of the phantom images. We removed low-frequency background trends, such as the heel-effect, by second-order polynomial two dimensional-surface correction. Regions of interest (ROIs) of 256 × 256 pixels were calculated using an ensemble average of half-overlapping segments (128 pixels in each direction) from the sub-images. In this way, 64 ROIs were used for each breast density. Finally, one-dimensional NPS values were obtained by averaging the central value ±the values of seven rows across the axis (excluding the axis itself) [44].

## Results

### Breast density-dependence of the detection rate

**Fig 5:**
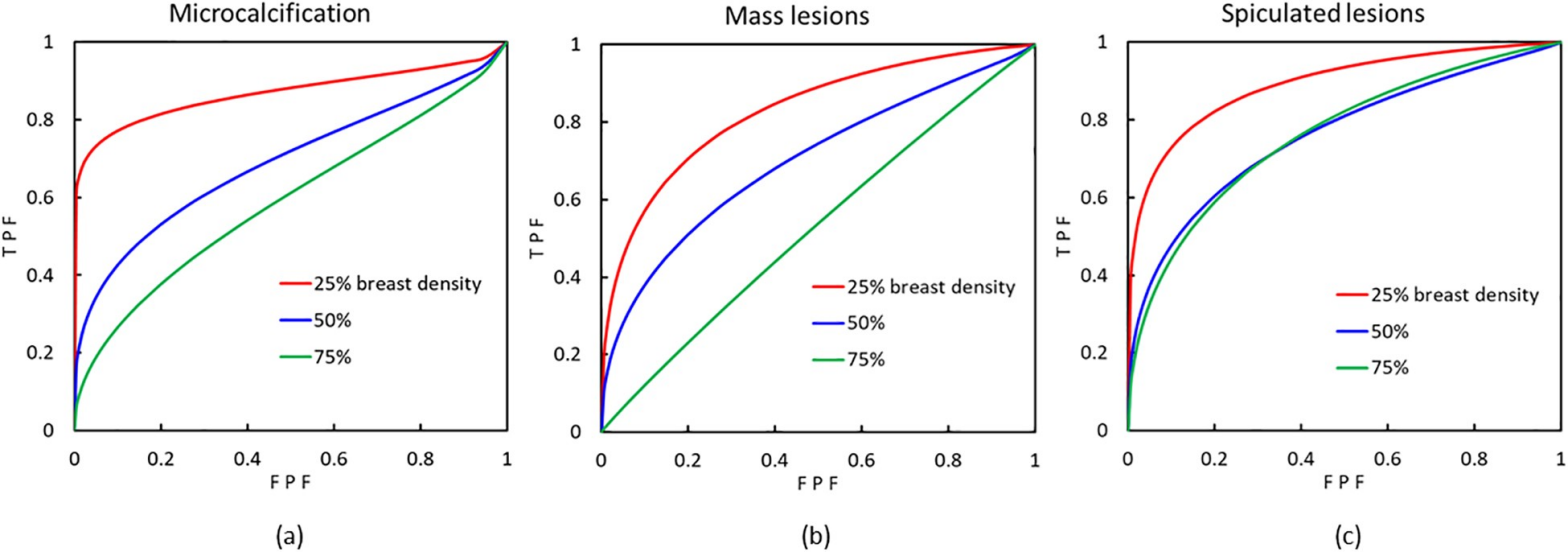
The relationship between the detection rate of lesions and breast density. (a), (b), and (c) represent microcalcification, mass lesions, and spiculated lesions, respectively. Each curve indicates an average of the results of the five observers. TPF, true positive fraction; FPF, false positive fraction.

Fig 5 depicts the influence of breast density on the detection rate of each lesion and Table 2 summarizes the results of the AUCs of all the above curves. As a whole, it seems that the lesion detection rate has a tendency to decrease with an increase in breast density. For instance, the percentage decrease in AUC for microcalcifications for 50% and 75% breast density were 23.7% and 33.3%, respectively, compared to the AUC for 25% breast density. Table 3 summarizes the results of Student’s t test (two-tailed with Bonferroni correction) for each combination conducted in this study. The only two combinations with no statistically significant difference in the detection rate were those of microcalcifications or spiculated lesions between phantoms with 50% and 75% breast densities.

**Table 2:** Area under the receiver operating characteristic curve for breast density and the detection rate for the three types of lesions.

	Breast density		
Type of lesion	25%	50%	75%
**Microcalcification**	0.860	0.656 (23.7)	0.574 (33.3)
**Mass lesion**	0.857	0.703 (18.0)	0.533 (37.8)
**Spiculated lesion**	0.910	0.778 (14.5)	0.770 (15.4)
**Average**	0.876	0.712 (18.7)	0.626 (28.5)

Each value is averaged for five observers. The number in parentheses indicates the percentage decrease compared to that of the 25% breast density phantom.

**Table 3:** Results of statistical analysis of the detection rate between breast densities.

Type of lesion	Between 25% and 50%		Between 50% and 75%		Between 25% and 75%	
	*P*	95% CI	*P*	95% CI	*P*	95% CI
**Microcalcification**	0.0144*	0.0765–0.3331	0.1787	-0.0576–0.2212	0.0003*	0.2167–0.3565
**Mass lesion**	0.0001*	0.1258–0.1822	0.0058*	0.0822–0.2582	0.0003*	0.2453–0.4031
**Spiculated lesion**	0.0465*	0.0033–0.2603	0.7595	-0.0553–0.0701	0.0093*	0.0570–0.2214
**Average**	0.0039*	0.0880–0.2390	0.0113*	0.0325–0.1405	0.0000*	0.2144–0.2856

All *p* values were calculated using Student’s t test. The 95% CI is that of the difference in AUC between the breast densities being compared for each lesion.

CI, confidence interval; AUC, area under the receiver operating characteristic curve.

* *p* < 0.0500.

### Noise distribution of the phantom image

**Fig 6:**
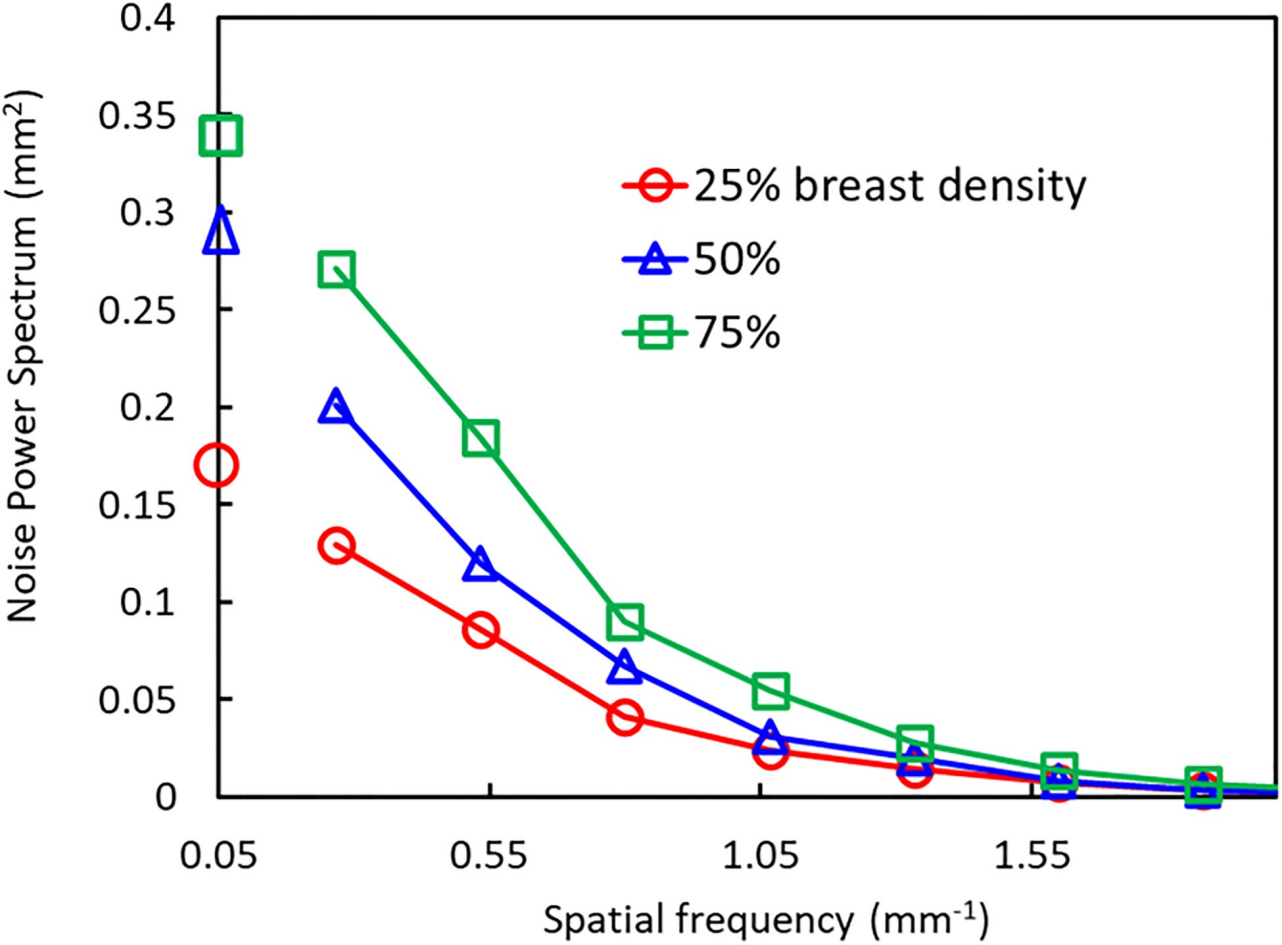
Noise-power spectra (NPS) and spatial frequency for three different breast densities of the original phantom. The circles, triangles, and squares represent breast densities of 25%, 50%, and 75%, respectively. The spectral values are extrapolated to a spatial frequency of 0.05 mm^-1^, which corresponds to the frequency band of the spiculated lesions used in this study.

Fig 6 depicts the relationship between the NPS and spatial frequency for each breast density. At a frequency of 0.05 mm^-1^, the spectral value for 25% breast density is predicted to be lower than that for 50% and 75% densities. However, the difference in spectral value was smaller between breast densities of 50% and 75%.

## Discussion

Even if the accurate volumetric breast density measurement is established, individualized screening mammography cannot be achieved unless the effect of breast density on the risk of missing lesions is elucidated. In the mammographic sensitivity measurement using actual clinical images, many factors described in the introduction section are intricately intertwined in addition to the breast density. We think this is the main reason why the mammographic sensitivity for dense breasts differs significantly within studies. Therefore, we performed the ROC-phantom study by five certified radiological technologists in screening mammography to investigate the impact only of breast density. All of them have trained using a special program regarding mammographic technique, reading, and quality control on screening mammography conducted by Japan Central Organization on Quality Assurance of Breast Cancer Screening, and has passed rigorous examinations including clinical reading. In addition, they routinely observe all the mammograms carefully, and have been entrusted from the certified radiologists to decide whether to add a magnified imaging for abnormal shadows. Thus, we believe it is significant that the certified radiological technologists participated for this study.

Most of the results of this study were in accordance with the current understanding of the inverse relationship between the detection rate of a lesion and breast density. Mandelson et al. showed that the percentage decrease of mammographic sensitivity for dense breasts to that of fatty breasts was 62% [45], whereas as can be observed in Table 2, the percentage decrease of AUC for 75% breast density to that for 25% is 37.8% in maximum. The difference of the percentage decrease between this study and Mandelson et al. as 24.2% may be due to some factors other than breast density described in the introduction section such as imaging technology etc., and because they used interval cancer to determine the mammographic sensitivity. Other literature also show the higher percentage decrease of mammographic sensitivity for dense breasts to fatty breasts compared with our phantom study (Mousa et al; 34.3~62.5% [46] Ekpo et al; 22.1~70.0% [21]). We believe that it is important to understand the effect of breast density alone from various intertwined factors in order to make a countermeasure for missing lesions.

In this study, an inverse relationship between the detection rate of a lesion and breast density was detected between all breast densities for the detection of mass lesions, in particular. As can be observed in Fig 5 and Table 3, however, for spiculated lesions, which are larger and exhibit low contrast, as well as for microcalcifications, which are very small and exhibit low contrast, the influence by breast density is not as simple. For spiculated lesions, we hypothesized that this phenomenon was due to characteristics of the spatial frequencies of the simulated lesion, and distribution of the fibroglandular-equivalent tissue. The relationship between the NPS and spatial frequency for different breast densities, depicted in Fig 6, was in accordance with results of the ROC curve for spiculated lesions, which implies that certain object sizes, e.g., those of 10 mm in diameter, are likely to be missed, depending on breast density.

On the other hand, the detection rate of microcalcifications decreased rapidly between breast densities of 25% and 50%, but not as rapidly at a higher breast density. From this phenomenon, we hypothesize that there may be a threshold for detection of microcalcifications. The non-linear relationship between breast density and the missed lesion rate, depending on the lesion type, should be taken into account when considering the influence of breast density in individualized screening mammography.

The current study had several limitations. First, we simulated only three lesion types and three different breast densities. In clinical cases, lesions are much more varied in terms of shape, size, and contrast. Future studies will need to include many more lesion types and fibroglandular distributions, such as an increasing distribution towards the nipple, for more accurate quantification of the missed lesion risk. However, although only three lesion types were analyzed in this study, these are the most commonly encountered lesions in mammography. Second, observers in this study were only five certified radiological technologists. In future studies, ROC-observation should be performed with more observers in addition of certified radiologists. We believe that overcoming these limitations will enable the development of a new breast density classification map that relates the detailed breast density range to the missed lesion rate. Such a map could potentially replace the currently used four-class map of the BI-RADS.

In conclusion, an adjustable-density breast phantom, consisting of adipose- and fibroglandular-equivalent materials with consistent X-ray absorption characteristics over the entire mammographic energy range, was developed. An ROC study was performed for three types of lesions and three different breast densities, in order to quantify the relationship between the missed lesion rate and breast density. Although the detection rate tended to decrease with increasing breast density, it depended on the specific lesion type and breast density. Ultimately, we quantified the missed lesion risk in different densities of breast tissue, which is an important step towards implementing advanced individualized screening mammography.

## Conclusions

Our findings suggest that there is a non-linear relationship between breast density and the missed lesion rate. This relationship, discovered using a novel, adjustable-density breast phantom, will be useful in the development of individualized screening mammography, especially for individuals with dense breast tissue.

## Supporting information

S1 Fig The relationship between the detection rate of lesions and the certification level of the observers. From the areas under the receiver operating characteristic curves, more experience leads to a higher detection rate. Each curve indicates an average of the three types of lesions in 25% breast density. TPF, true positive fraction; FPF, false positive fraction. Dependence of the detectability on the observer for 25% breast density are indicated in S1 Fig and S1 Table, but involves no statistical analysis owing to the presentation of the results for each observer. Tendency of the lesion detection was higher for observers with a higher certification level in clinical mammography. This tendency was similar for the other breast densities. Accordingly, although there is no statistical evidence, this was observed to be relevant between the lesion detection rate and the level of certification of the observer. This indicated that the original phantom might be used to carry out an ROC study, as it represents actual breast tissue.
(TIF)

S1 Table Area under the receiver operating characteristic curve for the lesion detection rate and certification level of the observer. (DOCX)

## References

[1] Nagini S. Breast cancer: current molecular therapeutic targets and new players. Anticancer Agents Med Chem. 2017;17: 152–163. 10.2174/1871520616666160502122724 27137076

[2] Akram M, Iqbal M, Daniyal M, Khan AU. Awareness and current knowledge of breast cancer. Biol Res. 2017;50: 33 10.1186/s40659-017-0140-9 28969709 PMC5625777

[3] Han SJ, Guo QQ, Wang T, Wang YX, Zhang YX, Liu F, et al Prognostic significance of interactions between ER alpha and ER beta and lymph node status in breast cancer cases. Asian Pac J Cancer Prev. 2013;14: 6081–6084. 10.7314/apjcp.2013.14.10.6081 24289629

[4] Siegel R, Naishadham D, Jemal A. Cancer statistics, 2013. CA Cancer J Clin. 2013;63: 11–30. 10.3322/caac.21166 23335087

[5] Marmot MG, Altman DG, Cameron DA, Dewar JA, Thompson SG, Wilcox M. The benefits and harms of breast cancer screening: an independent review. Lancet. 2012;380:1778–86. 10.1016/S0140-6736(12)61611-0 23117178

[6] Schapira MM, Hubbard RA, Seitz HH, Conant EF, Schnall M, Cappella JN, et al The impact of a risk-based breast cancer screening decision aid on initiation of mammography among younger women: report of a randomized trial. MDM Policy Pract. 2019;4: 1–13. 10.1177/2381468318812889 30729166 PMC6350139

[7] Siu AL. Screening for breast cancer: US Preventive Services Task Force recommendation statement. Ann Intern Med. 2016;164: 279–296. 10.7326/M15-2886 26757170

[8] Oeffinger KC, Fontham ET, Etzioni R, Herzig A, Michaelson JS, Shih YC, et al Breast cancer screening for women at average risk: 2015 guideline update from the American Cancer Society. JAMA. 2015;314: 1599–1614. 10.1001/jama.2015.12783 26501536 PMC4831582

[9] Lee CI, Chen LE, Elmore JG. Risk-based Breast Cancer Screening: Implications of Breast Density. Med Clin North Am. 2017;101: 725–741. 10.1016/j.mcna.2017.03.005 28577623 PMC5458625

[10] Von Euler-Chelpin M, Lillholm M, Vejborg I, Nielsen M, Lynge E. Sensitivity of screening mammography by density and texture: a cohort study from a population-based screening program in Denmark. Breast Cancer Res. 2019;21: 111 10.1186/s13058-019-1203-3 31623646 PMC6796411

[11] Posso M, Louro J, Sánchez M, Román M, Vidal C, Sala M, et al Mammographic breast density: how it affects performance indicators in screening programmes? Eur J Radiol. 2019;110: 81–87. 10.1016/j.ejrad.2018.11.012 30599878

[12] Vinnicombe SJ. Breast density: why all the fuss? Clin Radiol. 2018;73: 334–357. 10.1016/j.crad.2017.11.018 29273225

[13] Théberge I, Guertin MH, Vandal N, Côté G, Dufresne MP, Pelletier É, et al Screening sensitivity according to breast cancer location. Can Assoc Radiol J. 2019;70: 186–192. 10.1016/j.carj.2018.10.007 30853307

[14] Weigel S, Heindel W, Heidrich J, Hense HW, Heidinger O. Digital mammography screening: sensitivity of the programme dependent on breast density. Eur Radiol. 2017;27: 2744–2751. 10.1007/s00330-016-4636-4 27822617

[15] Saarenmaa I, Salminen T, Geiger U, Heikkinen P, Hyvärinen S, Isola J, et al The effect of age and density of the breast on the sensitivity of breast cancer diagnostic by mammography and ultasonography. Breast Cancer Res Treat. 2001;67: 117–123. 10.1023/a:1010627527026 11519860

[16] Spalluto LB, Roumie CL, Bonnett KR, Schlundt DG, DeBenedectis CM, Wilkins CH. Women’s response to state-mandated language in dense breast notification. Breast J. 2018;24: 1046–1050. 10.1111/tbj.13119 30255589 PMC6239956

[17] Houssami N, Lee CI. The impact of legislation mandating breast density notification—review of the evidence. Breast. 2018;42: 102–112. 10.1016/j.breast.2018.09.001 30236594 PMC6487858

[18] Horný M, Shwartz M, Duszak R Jr, Christiansen CL, Cohen AB, Burgess JF Jr. Characteristics of state policies impact health care delivery: an analysis of mammographic dense breast notification and insurance legislation. Med Care. 2018;56: 798–804. 10.1097/MLR.0000000000000967 30036236

[19] Gunn CM, Kressin NR, Cooper K, Marturano C, Freund KM, Battaglia TA. Primary care provider experience with breast density legislation in Massachusetts. J Womens Health. 2018;27: 615–622. 10.1089/jwh.2017.6539 29338539 PMC7061298

[20] Chau SL, Alabaster A, Luikart K, Brenman LM, Habel LA. The effect of California’s breast density notification legislation on breast cancer screening. J Prim Care Community Health. 2017;8: 55–62. 10.1177/2150131916674889 27799412 PMC5932660

[21] https://www.qabcs.or.jp/archives/001/201703/170321_1.pdf.

[22] Yamamuro M, Asai Y, Yamada K, Ozaki Y, Matsumoto M, Murakami T. Prediction of glandularity and breast radiation dose from mammography results in Japanese women. Med Biol Eng Comput. 2019;57: 289–298. 10.1007/s11517-018-1882-4 30099671

[23] Chiu SYH, Duffy S, Yen AMF, Tabár L, Smith RA, Chen HH. Effect of baseline breast density on breast cancer incidence, stage, mortality, and screening parameters: 25-year follow-up of a Swedish mammographic screening. Cancer Epidemiol Biomarkers Prev. 2010;19: 1219–1228. 10.1158/1055-9965.EPI-09-1028 20406961

[24] Ekpo EU, Alakhras M, Brennan P. Errors in mammography cannot be solved through technology alone. Asian Pac J Cancer Prev. 2018;19: 291–301. 10.22034/APJCP.2018.19.2.291 29479948 PMC5980911

[25] Hofvind S, Geller BM, Skelly J, Vacek PM. Sensitivity and specificity of mammographic screening as practised in Vermont and Norway. Br J Radiol. 2012;85: e1226–e1232. 10.1259/bjr/15168178 22993383 PMC3611728

[26] Mandelson MT, Oestreicher N, Porter PL, White D, Finder CA, Taplin SH, et al Breast density as a predictor of mammographic detection: comparison of interval- and screen-detected cancers. J Natl Cancer Inst. 2000;92: 1081–1087. 10.1093/jnci/92.13.1081 10880551

[27] Buist DSM, Porter PL, Lehman C, Taplin SH, White E. Factors contributing to mammography failure in women aged 40–49 years. J Natl Cancer Inst. 2004;96: 1432–1440. 10.1093/jnci/djh269 15467032

[28] Hollingsworth AB. Redefining the sensitivity of screening mammography: A review. Am J Surg. 2019;218: 411–418. 10.1016/j.amjsurg.2019.01.039 30739738 PMC6640096

[29] Bouwman RW, Van Engen RE, Young KC, den Heeten GJ, Broeders MJM, Schopphoven S, et al Average glandular dose in digital mammography and digital breast tomosynthesis: comparison of phantom and patient data. Phys Med Biol. 2015;60: 7893–7907. 10.1088/0031-9155/60/20/7893 26407015

[30] Bouwman RW, Van Engen RE, Young KC, Veldkamp WJ, Dance DR. Dose assessment in contrast enhanced digital mammography using simple phantoms simulating standard model breasts. Phys Med Biol. 2014;60: N1–N7. 25500435 10.1088/0031-9155/60/1/N1

[31] Izdihar K, Kanaga KC, Krishnapillai V, Sulaiman T. Determination of tube output (kVp) and exposure mode for breast phantom of various thicknesses/glandularity for digital mammography. Malays J Med Sci. 2015;22: 40–49. PMC4390773 25892949

[32] Waal D, Emaus MJ, Bakker MF, Heeten GJ, Karssemeijer N, Pijnappel RM, et al Geographic variation in volumetric breast density between screening regions in the Netherlands. Eur Radiol. 2015; 25: 3328–37. 10.1007/s00330-015-3742-z 26134996 PMC4595533

[33] https://www.mammaria.jp/dense_breast/.

[34] Young KC, Burch A, Oduko JM. Radiation doses received in the UK Breast Screening Programme in 2001 and 2002. Br J Radiol. 2005;78: 207–218. 10.1259/bjr/41095952 15730985

[35] Nishide H, Ohta K, Murata K, Kodera Y. Exposure conditions according to breastthickness and glandularity in Japanese Women In: Tingberg A, Lång K, Timberg P, editors. Breast Imaging. IWDM 2016. Lecture Notes in Computer Science, 9699; 2016 pp. 408–414.

[36] Beckett JR, Kotre CJ. Dosimetric implications of age related glandular changes in screening mammography. Phys Med Biol. 2000;45: 801–813. 10.1088/0031-9155/45/3/316 10730972

[37] ICRU Report 79. Receiver operating characteristic analysis in medical imaging. ICRU (International Commission on Radiation Units and Measurements) 2008; Oxford University Press, Oxford, UK.

[38] ICRU Report 44. Tissue substitutes in radiation dosimetry and measurement. ICRU (International Commission on Radiation Units and Measurements) 1989; Bethesda, MD.

[39] Berger MJ, Hubbell J, Seltzer SM, Chang J, Coursey JS, Sukumar R, et al. Photon Cross Sections Database, NIST Standard Reference Database 8 (XGAM). https://physics.nist.gov/PhysRefData/Xcom/Text/version.shtml.

[40] Eng J. ROC analysis: web-based calculator for ROC curves Baltimore: Johns Hopkins University http://www.jrocfit.org.

[41] Jankowski KRB, Flannelly KJ, Flannelly LT. The t-test: an influential inferential tool in chaplaincy and other healthcare research. J Health Care Chaplain. 2018;24: 30–39. 10.1080/08854726.2017.1335050 28622103

[42] Kim TK. T test as a parametric statistic. Korean J Anesthesiol 2015;68: 540–546. 10.4097/kjae.2015.68.6.540 26634076 PMC4667138

[43] Ichikawa K, Kodera Y, Nishimura A, Hasegawa M, Kimura N, Takemura A, et al Analysis method of noise power spectrum for medical monochrome liquid crystal displays. Radiol Phys Technol. 2008;1: 201–207. 10.1007/s12194-008-0029-y 20821148

[44] IEC (International Electrotechnical Commission). IEC 62220-1-1:2015. Medical electrical equipment—Characteristics of digital X-ray imaging devices—Part 1–1: Determination of the detective quantum efficiency—Detectors used in radiographic imaging. Geneva, Switzerland: IEC, 2015.

[45] Mandelson MT, Oestreicher N, Porter PL, White D, Finder CA, Taplin SH, et al Breast density as a predictor of mammographic detection: comparison of interval- and screen-detected cancers. J Natl Cancer Inst. 2000;92: 1081–1087. 10.1093/jnci/92.13.1081 10880551

[46] Mousa DSAL Ryan EA, Thoms CM Brennan PC. What effect does mammographic breast density have on lesion detection in digital mammography? Clin Radiol. 2014;69: 333–341. 10.1016/j.crad.2013.11.014 24424328

