# Supplementary figures and images for: The effect of breast density on the missed lesion rate in screening digital mammography determined using an adjustable-density breast phantom tailored to Japanese women
Source: PLoS One. 2021 Jan 7;16(1):e0245060. doi: 10.1371/journal.pone.0245060 (PMC7790234; doi:10.1371/journal.pone.0245060)

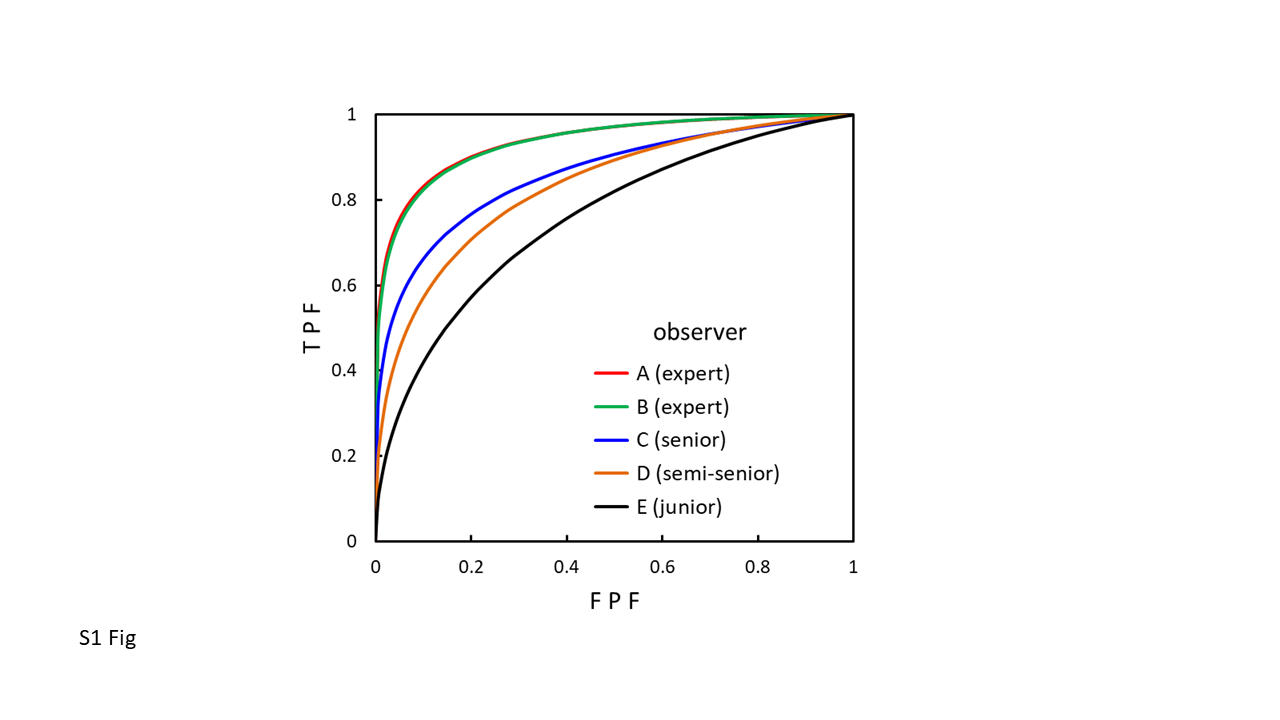

Supplement: S1 Fig — From the areas under the receiver operating characteristic curves, more experience leads to a higher detection rate. Each curve indicates an average of the three types of lesions in 25% breast density. TPF, true positive fraction; FPF, false positive fraction. Dependence of the detectability on the observer for 25% breast density are indicated in S1 Fig and S1 Table, but involves no statistical analysis owing to the presentation of the results for each observer. Tendency of the lesion detection was higher for observers with a higher certification level in clinical mammography. This tendency was similar for the other breast densities. Accordingly, although there is no statistical evidence, this was observed to be relevant between the lesion detection rate and the level of certification of the observer. This indicated that the original phantom might be used to carry out an ROC study, as it represents actual breast tissue. (TIF) [file pone.0245060.s001.TIF]
