# Supplementary material for: The effect of breast density on the missed lesion rate in screening digital mammography determined using an adjustable-density breast phantom tailored to Japanese women
Source: PLoS One. 2021 Jan 7;16(1):e0245060. doi: 10.1371/journal.pone.0245060 (PMC7790234; doi:10.1371/journal.pone.0245060)
Supplement: S1 Table — (DOCX) [file pone.0245060.s002.docx]

**S1 Table. Area under the receiver operating characteristic curve for the lesion detection rate and certification level of the observer.**

|  |  | **Breast density** | | |
| --- | --- | --- | --- | --- |
| **Observer** | **Certification level** | **25%** | **50%** | **75%** |
| A | Expert | 0.938 | 0.790 | 0.690 |
| B | Expert | 0.936 | 0.765 | 0.654 |
| C | Senior | 0.860 | 0.744 | 0.622 |
| D | Semi-senior | 0.831 | 0.691 | 0.586 |
| E | Junior | 0.755 | 0.604 | 0.575 |

Each value is averaged for three types of lesions (microcalcifications, mass lesions, and spiculated lesions).
